# Supplementary material for: Formulation of Bioerodible Ketamine Microparticles as an Analgesic Adjuvant Treatment Produced by Supercritical Fluid Polymer Encapsulation
Source: Pharmaceutics. 2018 Dec 6;10(4):264. doi: 10.3390/pharmaceutics10040264 (PMC6321204; doi:10.3390/pharmaceutics10040264)
Supplement: Supplementary file 1 [file pharmaceutics-10-00264-s001.pdf]

Supplementary Information:

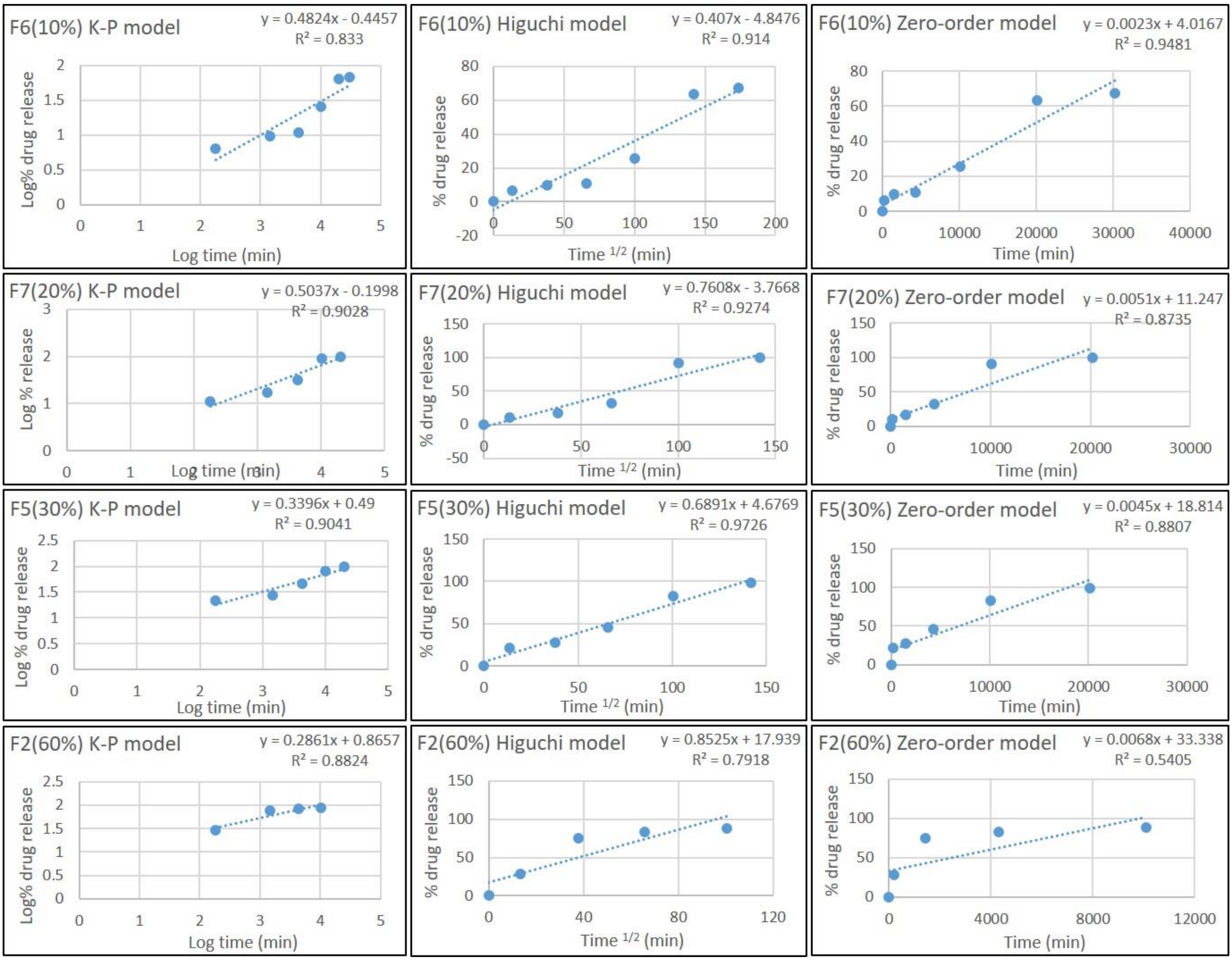

Figure S1. Drug release kinetics plots of F2, F5, F6 and F7.

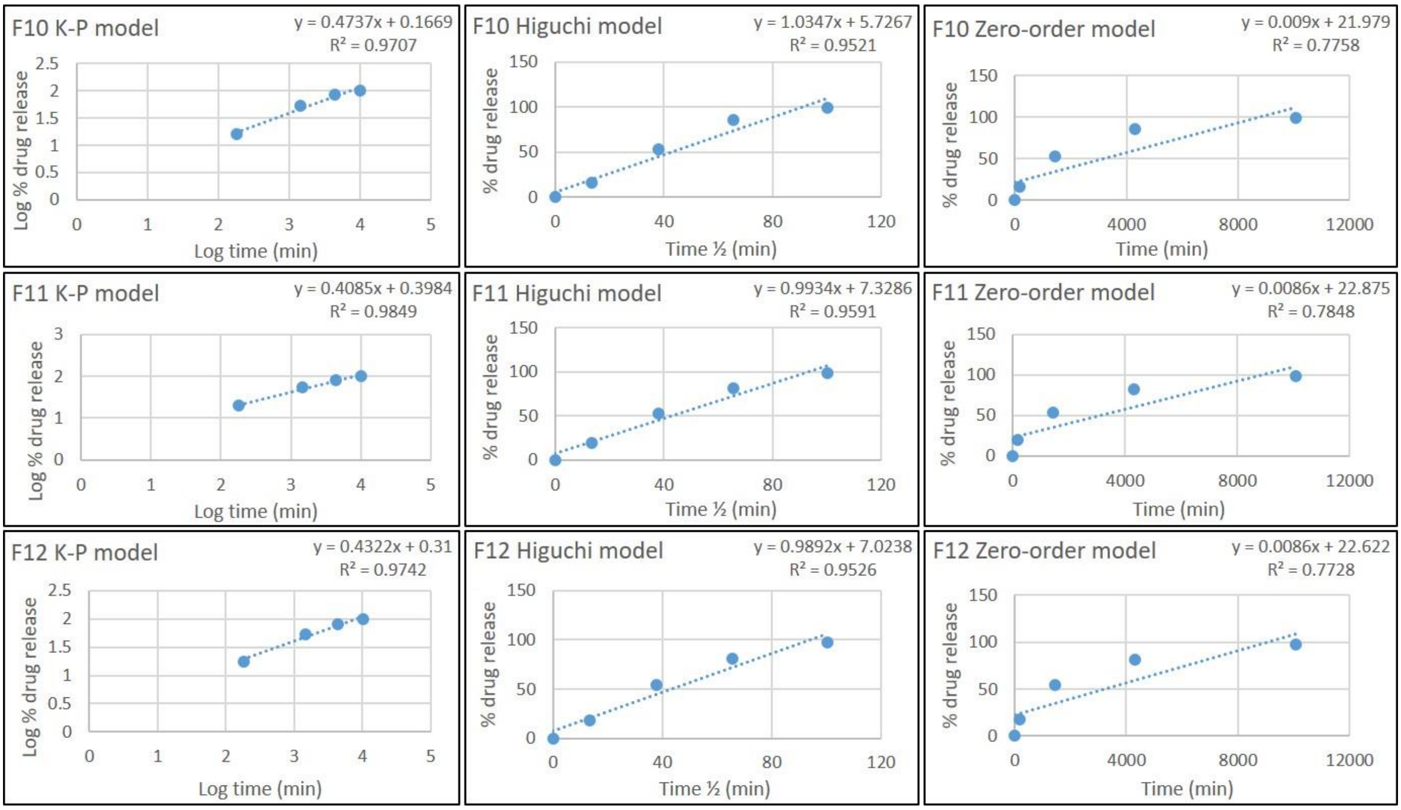

Figure S2. Drug release kinetics plots of F10, F11 and F12.

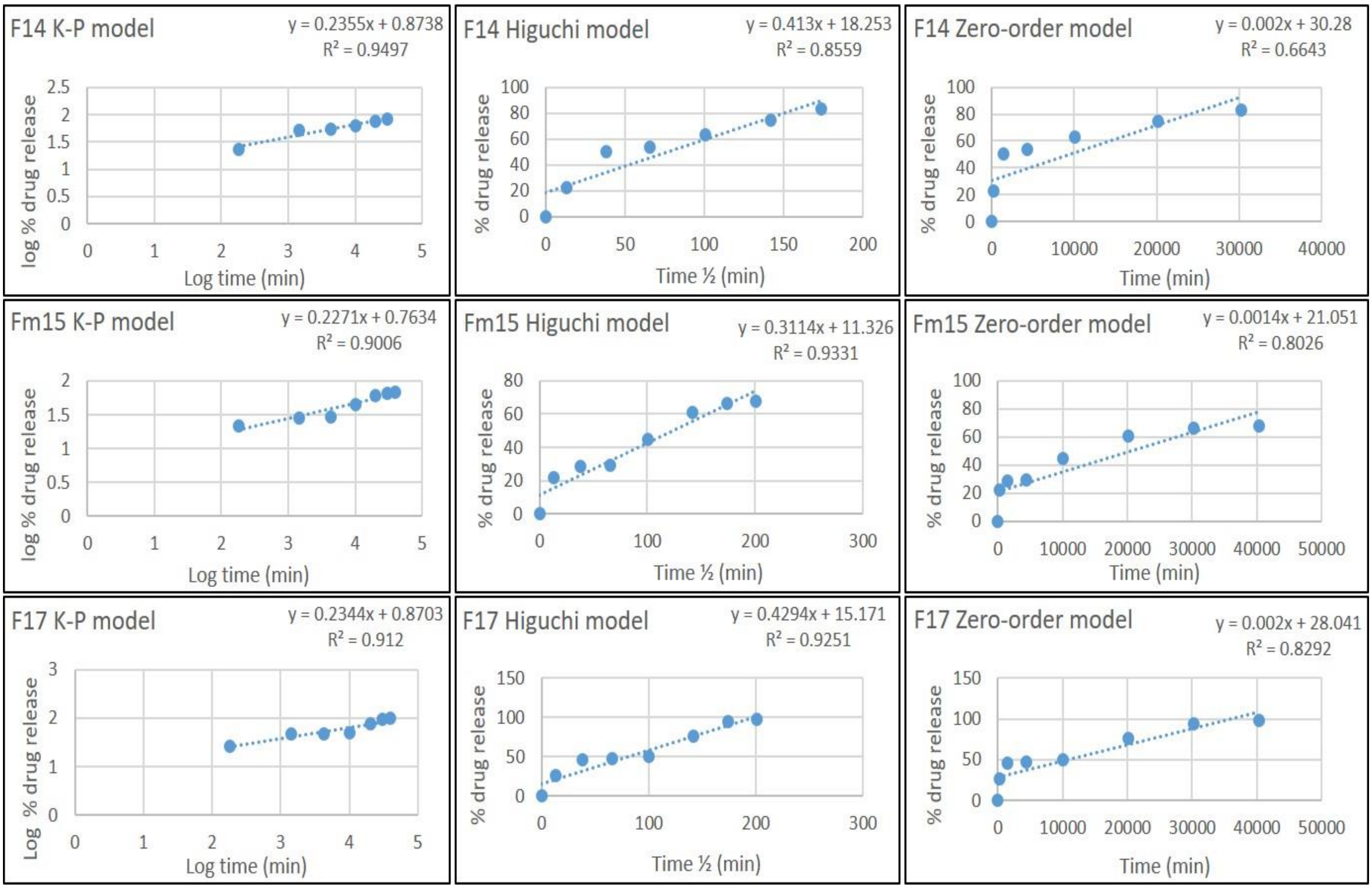

Figure S3. Drug release kinetics plots of F14, F15 and F17.

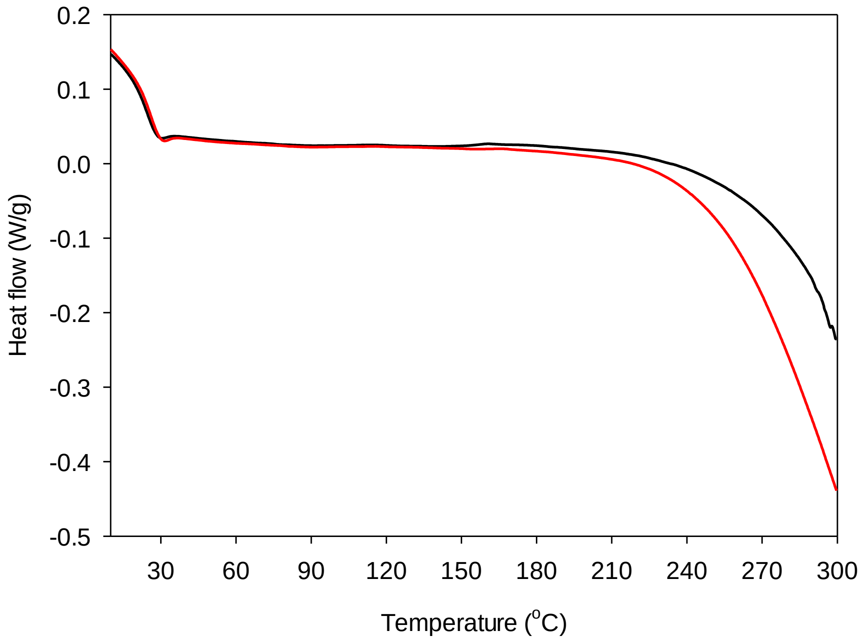

Figure S4. DSC curves of PLGA5050-1.5E before and after loading with ketamine. The curves displayed above are the 2nd heating. Heating rate is 10 °C/min from 0 to 300 °C.

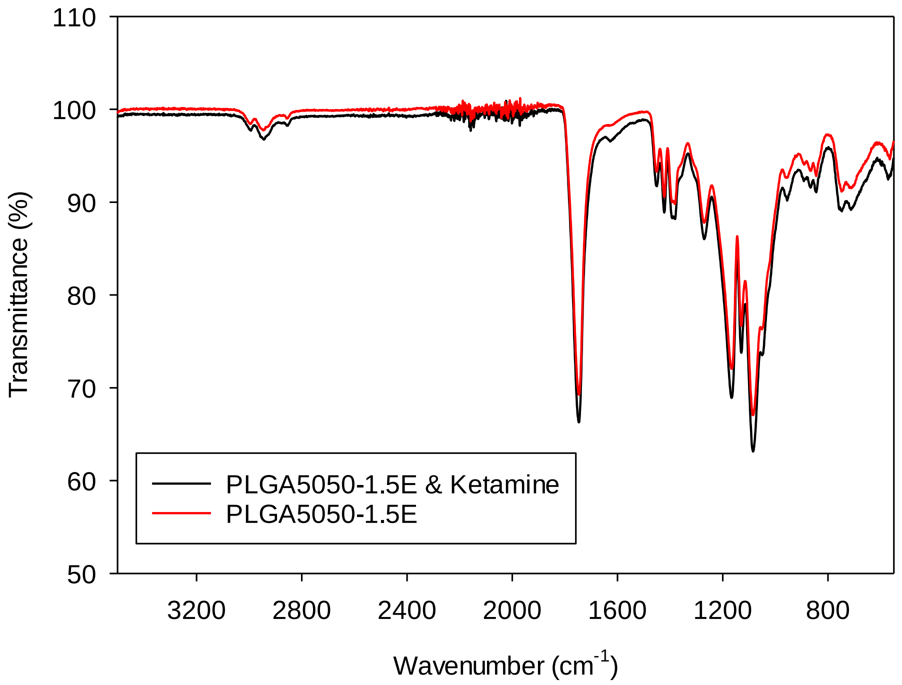

Figure S5. FTIR spectra of PLGA5050-1.5E before and after loading with ketamine.
